# Phylogeography, Salinity Adaptations and Metabolic Potential of the Candidate Division KB1 Bacteria Based on a Partial Single Cell Genome

**DOI:** 10.3389/fmicb.2016.01266

**Published:** 2016-08-22

**Authors:** Lisa M. Nigro, Andrew S. Hyde, Barbara J. MacGregor, Andreas Teske

**Affiliations:** Department of Marine Sciences, University of North Carolina at Chapel HillChapel Hill, NC, USA

**Keywords:** Candidate Division KB1, Orca Basin, phylogeography, genome, hypersaline

## Abstract

Deep-sea hypersaline anoxic basins and other hypersaline environments contain abundant and diverse microbial life that has adapted to these extreme conditions. The bacterial Candidate Division KB1 represents one of several uncultured groups that have been consistently observed in hypersaline microbial diversity studies. Here we report the phylogeography of KB1, its phylogenetic relationships to Candidate Division OP1 Bacteria, and its potential metabolic and osmotic stress adaptations based on a partial single cell amplified genome of KB1 from Orca Basin, the largest hypersaline seafloor brine basin in the Gulf of Mexico. Our results are consistent with the hypothesis – previously developed based on ^14^C incorporation experiments with mixed-species enrichments from Mediterranean seafloor brines – that KB1 has adapted its proteins to elevated intracellular salinity, but at the same time KB1 apparently imports glycine betaine; this compatible solute is potentially not limited to osmoregulation but could also serve as a carbon and energy source.

## Introduction

Hypersaline environments are abundant on Earth (e.g., Boetius and Joye, 2009) and include hypersaline lakes, such as the Dead Sea or the Great Salt Lake; evaporative lagoons; salterns; and hypersaline marine basins where salt deposits dissolve into deep-sea water and create distinct “lakes” of high-density brines on the seafloor of the Red Sea, Mediterranean Sea and Gulf of Mexico. These deep-sea hypersaline anoxic basins (DHABs) have been the recent focus of molecular microbial investigation (Eder et al., 2001; van der Wielen, 2005; La Cono et al., 2011; Yakimov et al., 2013). In spite of their harsh conditions, 16S rRNA gene sequencing surveys indicate that abundant and diverse microbial life has adapted to these extreme environments. Several uncultured groups were first observed in DHAB microbial diversity studies, including the bacterial Candidate Division KB1, the archaeal Mediterranean Seafloor Brine Lake Group 1 (MSBL-1), and other ‘MSBL’ groups within the Phylum Proteobacteria. Since these uncultured, presumably halophilic groups mostly lack genomic analysis (see Mwirichia et al., 2016 for a recent exception), their physiology and survival strategies have not been described (Antunes et al., 2011).

High-salinity environments are particularly challenging to microorganisms because biological membranes are sensitive to osmotic pressure, requiring cells to maintain a cytoplasmic solute concentration higher than that of the surrounding brine in order to prevent water loss. There are two known strategies microorganisms use to prevent dehydration: the “salt in” strategy, where organisms actively pump salt into the cell (usually K^+^ and Cl^-^) and the contrasting “salt out” strategy, where organisms produce compatible organic solutes or transport them into the cytoplasm (Oren, 2008). It is also possible to utilize a combination of strategies (e.g., Lai and Gunsalus, 1992). Microorganisms that predominantly use the “salt in” strategy also require special enzymatic adaptations, namely a higher proportion of acidic amino acids and a lower proportion of hydrophobic amino acids. This same bias prevents normal enzymatic function at lower salinities and, therefore, “salt in” strategists have been documented to be specialists and true halophiles. On the other hand, organisms predominantly using the “salt out” strategy produce and/or import organic-compatible solutes that do not require special intracellular protein adaptations (Oren, 2008). Compatible solute production or transport can be regulated by the cell, allowing “salt out” strategists to live at a range of salinities. However, compatible solute production is more energetically costly than maintaining high intracellular salt concentrations (Oren, 1999).

Of particular interest are the Candidate Division KB1 bacteria, commonly found in high-salinity anoxic environments. KB1 was initially detected by 16S rRNA gene sequencing from the Kebrit Deep brine basin in the Red Sea (Eder et al., 1999). The uncultured KB1 bacteria were proposed to be responsible for assimilation of glycine betaine as carbon substrate. Enrichments from Mediterranean seafloor brines (Lake Médée) contained a mixed microbial community that predominately yielded 16S rRNA sequences associated with KB1 and the archaeal halophile MSBL1. The enrichment incorporated high concentrations of ^14^C-labeled glycine betaine, which resulted in increased concentrations of trimethylamine in the media, suggesting possible cleavage of glycine betaine into acetate and trimethylamine (Yakimov et al., 2013).

Here we report the phylogeography of KB1, its phylogenetic relationships to Candidate Division OP1 Bacteria, and its potential metabolic roles and osmotic stress strategies based on a partial single cell amplified genome (SAG) from Orca Basin, the largest hypersaline seafloor brine basin in the Gulf of Mexico (Shokes et al., 1977; Pilcher and Blumstein, 2007). Our results support the hypothesis that KB1 in Orca Basin has adapted its proteins to elevated salinity consistent with a “salt in” strategy; its genome also encodes an uptake system for glycine betaine, which might be used not just for osmoregulation but also possibly as a carbon and energy source.

## Materials and Methods

### Sample Collection and Processing

Brine samples from the ca. 200 m thick brine layer in the southern Orca Basin were collected in April 2012 onboard the R/V *Pelican* (Expedition PE12-22) at Orca Basin (Latitude 26.54.67 N, Longitude 91.21.65 W, 2410 mbsf) with Niskin bottles attached to a rosette sampler equipped with a CTD. Brine was transferred to an argon-flushed sterile canning jar and aliquoted into an N_2_-flushed sterile stoppered serum vial. The brine sample was stored at 4°C and sent to the Bigelow Single Cell Genomics Center (East Boothbay, Boothbay, ME, USA). The sample was processed for single cell sorting followed by multiple displacement amplification of cellular DNA and PCR screening of bacterial 16S rRNA genes (Martinez-Garcia et al., 2012; Stepanauskas, 2012). A cell identified as belonging to the KB1 group by 16S rRNA gene screening was selected for further genome amplification and sequencing.

### Genome Analysis

The assembled genome was uploaded and analyzed with the RAST (Aziz et al., 2008) and IMG/ER (Markowitz et al., 2012) annotation servers. Selected putative protein-encoding genes were further investigated by BLASTP searching the NCBI non-redundant protein database (Altschul et al., 1990). Protein sequences were also acquired for comparison through Uniprot^1^ and RAST (Aziz et al., 2008). Amino acids were aligned using MUSCLE (Edgar, 2004). The model selection tool was utilized in MEGA6 and trees were constructed with the maximum likelihood method (Tamura et al., 2013). Neighbor Joining trees (in Supplementary Material) were constructed using a Poisson correction with 1000 bootstrap replicates using MEGA6 (Tamura et al., 2013). Theoretical average protein isoelectric point analysis was performed by importing putative protein encoding genes from RAST into ExPASy’s Compute PI/Mw tool^2^. GC profiles were determined and plotted as described previously (Gao and Zhang, 2006).

### 16S rRNA Phylogenetic Analysis

Sequences classified as KB1 (which includes both the KB1 and OP1 Candidate Divisions) in the SILVA non-redundant database (ver. 119) (Pruesse et al., 2007) were analyzed with the ARB phylogenetic software package (Ludwig et al., 2004) using RaxML maximum likelihood (Stamatakis, 2006).

### Database Access

The SAG sequence is available in RAST (Genome ID 6666666.48888) and IMG/ER (IMG genome ID 2654588133).

## Results and Discussion

### Cell Sorting and Identification

A total of 32 single cells from the Orca Basin brine sample yielded bacterial 16S rRNA gene amplicons. 16 cells represented the KB1 group, 13 were identified as Deltaproteobacteria, 2 were assigned to the candidate phylum OD1 (Elshahed et al., 2005) and one to the Bacteroidetes phylum. These taxa were also observed in a Sanger 16S rRNA gene clone library from a previous research collection (Supplementary Figure S1). The KB1-associated SAG with the lowest Cp value (5:08 h), indicating the time the multiple displacement amplification reaches half-maximum fluorescence, was selected for genome sequencing.

### Phylogenetics and Phylogeography of KB1

Conflicting taxonomic classification schemes have obscured the phylogenetic status of KB1 and the related OP1. The major taxonomic classifiers within SILVA (Ref 119) have assigned all OP1 and KB1-related organisms to one phylum, KB1, and undefined lower-ranking taxonomic categories. On the other hand, Greengenes (ver. 13.8) recently classified OP1 and KB1-related sequences into a single phylum, OP1 (McDonald et al., 2012), while maintaining distinct class levels of OP1, KB1, Acetothermia, and MSBL6. Acetothermia has also been suggested as a phylum name for the entire OP1/KB1 complex (Rinke et al., 2013), based on a reconstructed genome obtained from the metagenome of a geothermal acidic stream (Takami et al., 2012).

**FIGURE 1:**
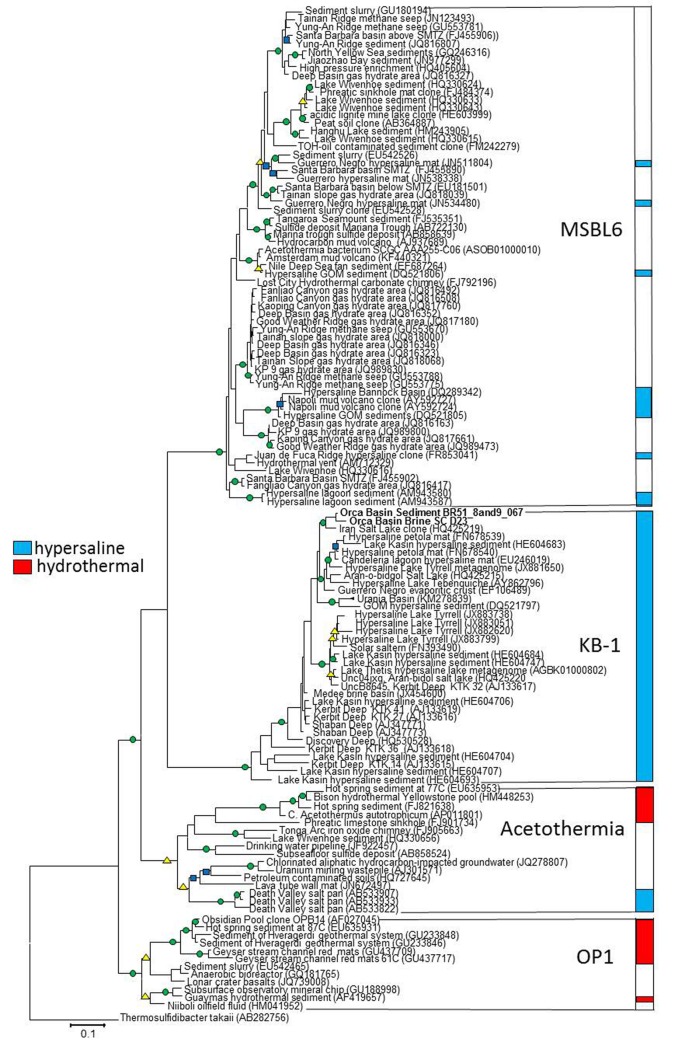
**RaxML tree of nearly full-length 16S rRNA genes of Candidate division KB1 and related bacteria.** The GTRMix rate distribution model was used with the rapid bootstrap algorithm. Circles indicate bootstrap support of 1000 replicates, green 90–100%, yellow 80–90% and blue 70–79%. The tree is rooted with the deeply branching bacterium *Thermosulfidibacter tokaii*, Group names indicate Greengenes Class taxonomy. The scale bar indicates the length of 0.1 nucleotide substitutions per site.

Our phylogenetic analyses of related sequences from the most recent non-redundant SILVA near-full length 16S rRNA gene database (version 119; Quast et al., 2013) found four distinct phylogenetic groups near the KB1 and OP1 candidate phyla with high (>80%) bootstrap statistical support (**Figure 1**). The single-cell sequences from Orca Basin brine identified as KB1 in the SILVA 119 database formed a monophyletic group with the initial KB1 sequences from the Red Sea Kebrit Deep brine basin (Eder et al., 1999) and with sequences from a wide range of other hypersaline habitats, including high salinity microbial mats, hypersaline lakes, solar salterns, and other deep-sea brine basins (van der Wielen, 2005; Tkavc et al., 2011; Makhdoumi-Kakhki et al., 2012) (**Figure 1**).

The initial Obsidian Pool sequence OPB14 (AF027045) (Hugenholtz et al., 1998) formed a distinct monophyletic group with sequences from other high-temperature (e.g., NCBI accession EU645931) and hydrocarbon-influenced (e.g., EU542465) environments; this group constitutes the OP1 lineage sensu stricto (Hugenholtz et al., 1998). The uncultured *Candidatus* taxon Acetothermum autotrophicum, reconstructed by metagenomic analysis from an acidic hot spring community (Takami et al., 2012), belonged to a cluster separate from the Obsidian Pool OP1 clone sequences. This separate, monophyletic cluster includes sequences from both high-temperature and hydrocarbon-impacted environments, and constitutes the Acetothermia sensu stricto (**Figure 1**).

The fourth monophyletic cluster is termed the Mediterranean Seafloor Brine Lake 6 (MSBL6) group, since it was initially found in 16S rRNA gene sequencing surveys of Mediterranean brine basins (Daffonchio et al., 2006); the original sequence dataset is represented here by Bannock Basin clone DQ289342 (**Figure 1**). Since then, the MSBL6 lineage has been populated by sequences from hydrocarbon-impacted environments, including cold seeps, sediments near sulfate-methane transition zones, and a methane-influenced brackish lake from which a partial single-cell genome [SAG SCGCAAA255-C06] was obtained (Rinke et al., 2013).

### Genome Analysis of KB1

The Orca Basin KB1-classified SAG genome contained 709508 bp and was estimated to be 25% complete based on the presence of 107 genes identified to be present in 95% of bacterial genomes in the Comprehensive Microbial Resource database (Dupont et al., 2012; Supplementary Table 1). Sequences for at least 12 tRNAs were found. The GC content was 46.70% (Supplementary Figure S2). Putative genes were identified for vitamin and cofactor biosynthesis and metabolism, a complete acetyl-CoA (Wood–Ljungdahl) pathway, cell wall biosynthesis, RNA protein metabolism, DNA metabolism and repair, and fatty acid and lipid metabolism. Genes potentially related to oxidative stress response, heat and cold shock response, and glycine betaine transport were also identified (Supplementary Table 2).

### Osmotic Regulation

The osmotic regulation strategy of KB1 is of particular interest since the KB1 clade contains sequences exclusively from hypersaline environments. KB1 contains genes potentially coding for uptake of compatible solutes such as glycine betaine or proline betaine, and for uptake of K^+^ (Supplementary Table 2), indicating either intracellular accumulation of compatible solutes as a “salt out” strategist, accumulation of potassium salts as a “salt in” strategist, or a combination of both strategies (Oren, 2008).

**FIGURE 2:**
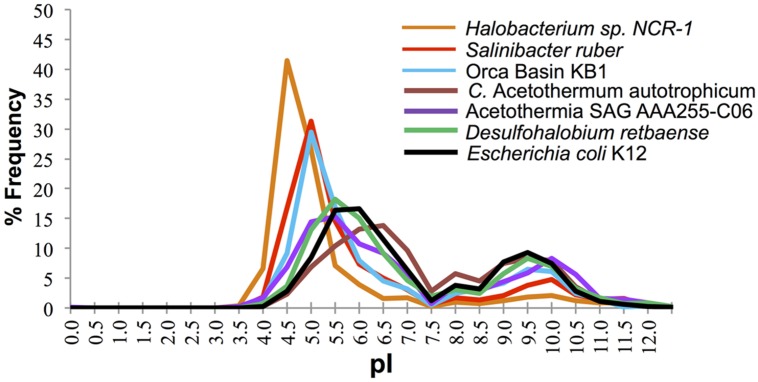
**Frequency of predicted isoelectric point (nearest 0.5) of putative proteins in the genomes of the OP1/KB1 phylum complex and other halophilic and non-halophilic bacteria.** The plot includes Orca Basin KB1; SAG SCGCAAA255-C06 from a methane and sulfide-rich zone of a brackish lake (Rinke et al., 2013), *C.* Acetothermum autotrophicum (Takami et al., 2012), *Halobacterium* NRC-1, a halophilic “salt in” archaeon; *Salinibacter ruber*, a “salt in” halophilic bacterium; *Desulfohalobium retbaense*, a “salt out” halophilic bacterium and *Escherichia coli* K12.

The distribution of predicted protein isoelectric points in the KB1 genome suggests that KB1 is adapted for using the “salt in” strategy (**Figure 2**). Accumulation of salts, primarily K^+^, for osmotic regulation was first observed in halophilic archaea in the phylum Halobacteria, and subsequently in the bacterium *Salinibacter ruber* in the phylum Bacteroidetes (Antón et al., 2002; Mongodin et al., 2005). The predicted proteins inferred from these organisms’ genomes contain a higher proportion of acidic amino acids than do proteins from microorganisms that do not accumulate intracellular salts (Mongodin et al., 2005). The isoelectric point distribution of inferred proteins from the KB1 SAG (**Figure 2**) indicated a preference for proteins with higher abundances of acidic amino acids (30% pI 5.0), similar to *S. ruber*, but proportionally fewer acidic amino acids than the extreme archaeal halophile, *Halobacterium* NCR-1 (~42% pI 4.5). Conversely, *C.* Acetothermum autotrophicum within the Acetothermia, and the partial SAG genome SCGC AAA255-C06 in the MSBL6 clade (Rinke et al., 2013), had protein isoelectric point abundance patterns similar to those of organisms that do not accumulate high concentrations of intracellular salts, including *Escherichia coli* and *Desulfohalobium retbaense*, a sulfate-reducing bacterium isolated from the hypersaline Retba Lake (Senegal, West Africa) thought to accumulate compatible solutes as its primary mechanism for osmoregulation (Spring et al., 2010).

**FIGURE 3:**
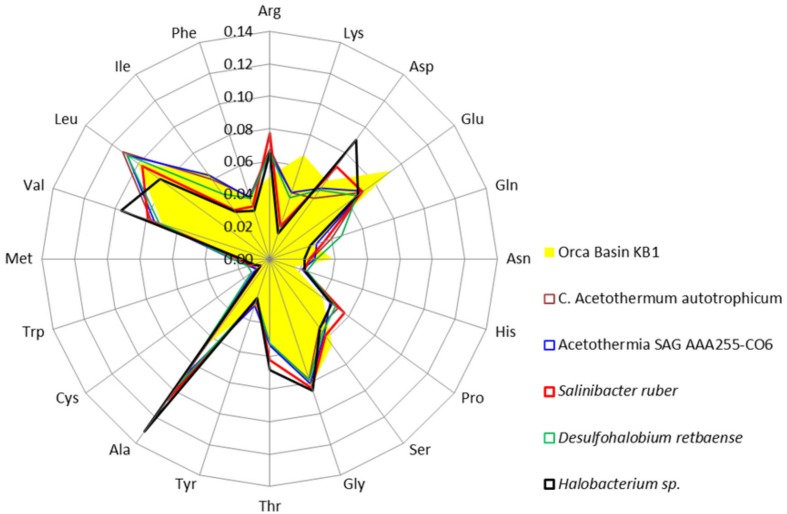
**Frequency of amino acids in putative proteins in the genomes of the OP1/KB1 phylum complex and other halophilic and non-halophilic bacteria.** The plot includes Orca Basin KB1; SAG SCGCAAA255-C06 from a methane- and sulfide-rich zone of a brackish lake (Rinke et al., 2013); *C.* Acetothermum autotrophicum (Takami et al., 2012); *Halobacterium* NRC-1, a halophilic “salt in” archaeon; *Salinibacter ruber*, a “salt in” halophilic bacterium; *Desulfohalobium retbaense*, a “salt out” halophilic bacterium.

Amino acids were further analyzed to determine which acidic amino acids (glutamate or aspartate) were contributing to the isoelectric point pattern in KB1. Analysis of individual amino acid abundances in proteins inferred from ORFs from the Orca Basin KB1 SAG indicated that glutamate was more abundant than aspartate, which was only marginally higher in abundance than in the non-halophilic or “salt out” strategist bacteria analyzed. However, bias for glutamate was not universal among the “salt in” strategists, as *Halobacterium* contained higher concentrations of aspartate, while *S. ruber* contained elevated acidic amino acids, but near-equal proportions of glutamate and aspartate (**Figure 3**).

### Utilization of Glycine Betaine in KB1

Glycine betaine is among the most common compatible solutes produced and/or imported by “salt out” strategists in response to osmotic stress. Glycine betaine transport systems can aid cells in maintaining osmotic balance and are well studied in cultured isolates, including non-halophilic *Escherichia* and *Salmonella* species which use these specific transport systems to adapt to elevated salinity within non-extreme survival limits (Perroud and Le Rudulier, 1985; Koo and Booth, 1994). Once in the cytoplasm, glycine betaine has several possible fates (Müller et al., 1981; Naumann et al., 1983; Möller et al., 1984; Heijthuijsen and Hansen, 1989; Watkins et al., 2014): it can be retained as a compatible solute, cleaved to acetate and trimethylamine (Naumann et al., 1983; Heijthuijsen and Hansen, 1989), or demethylated to dimethylglycine (Müller et al., 1981; Möller et al., 1984). Demethylation to glycine has been considered the dominant pathway in aerobic bacteria, while cleavage has been considered the dominant anaerobic pathway. However, demethylation does occur in some anaerobes, such as marine sulfate-reducing bacteria (van der Maarel et al., 1996) and marine methanogenic archaea (Watkins et al., 2014). In some methylotrophic methanogenic archaea, demethylation of glycine betaine to dimethylglycine has been observed to be connected directly to methanogenesis (Watkins et al., 2014).

The Orca Basin SAG KB1 contains a compact gene cassette potentially encoding a glycine betaine uptake system, specifically a 3-protein proline or glycine betaine ABC transporter system, ProU (genes *proVWX*) (Supplementary Figure S3). Neighbor-joining trees of the ProU proteins (ProV, ProW, ProX; Supplementary Figure S4), and bootstrap analysis of the tree topologies showed that these proteins did not form well-supported phylogenetic lineages with ProU protein sequences from known organisms. Interestingly, no recognizable glycine betaine uptake genes were observed in the nearly complete OP1-related genome of *C.* Acetothermum autotrophicum (Takami et al., 2012), indicating that glycine betaine uptake may not be universal to all members of the KB1 and OP1 clades. However, since the Orca Basin KB1 contains these genes, we propose that glycine betaine utilization starts with importing glycine betaine into the cell via the ATP binding cassette (ProU; T.C. 3.A.1.12.1).

Once glycine betaine is inside the cell, the genes detected near the transporter system suggest different possible fates. One possibility is demethylation and subsequent use of the methyl group as a carbon source via the acetyl-CoA (Wood–Ljungdahl) pathway. Investigating the physiological and phylogenetic diversity of methyltransferases, Ticak et al. (2014) recently provided evidence that a family of corrinoid-containing trimethylamine methyltransferases related to trimethylamine transferases in the methanogen *Methanosarcina* spp. demethylate glycine betaine in *Desulfitobacterium hafniense* Y51. The trimethylamine methyltransferase protein was upregulated during growth of *D. hafniense* on glycine betaine, and spectrophotometric evidence suggested that the recombinant protein converted glycine betaine and cob(l)alamin to dimethylglycine and methylcobalamin. Possibly, one or several putative methyltransferases in KB1 have a similar function (Supplementary Figure S5).

A KB1 methyltransferase (Supplementary Figure S3 and S5) could potentially transfer one of the methyl groups of glycine betaine to tetrahydrofolate, yielding dimethylglycine and 5-methyl-tetrahydrofolate. The fate of dimethylglycine remains unknown – no enzymes for dimethylglycine demethylation were found – but potential genes for glycine decarboxylase and sarcosine (N-methylglycine) oxidase were detected, indicating the possibility of complete demethylation to glycine (Supplementary Figure S7). The methylated tetrahydrofolate could possibly enter the methyl branch of the Wood–Ljungdahl pathway (Kreher et al., 2008; Ragsdale and Pierce, 2008), since the necessary enzymes appear to be encoded in the KB1 genome (Supplementary Figures S3 and S5; Supplementary Table 2). The pathway can, therefore, potentially be used in the oxidative direction if an appropriate electron acceptor (e.g., formate) is available and able to be utilized by the organism (Kreher et al., 2008). The carbon monoxide dehydrogenase complex and acetyl-CoA synthase were also detected in the partial KB1 genome. If the redox regime in the Orca Basin brine permits microorganisms to sustain the reductive acetyl-CoA pathway that reduces CO_2_ with formate or hydrogen, which is consistent with the thoroughly reducing conditions in surficial sediments in Orca Basin (Zhuang et al., 2016), the genomic evidence would allow the interpretation that glycine betaine provides KB1 with a source of C1 compounds for biosynthetic purposes (Supplementary Figure S6). Otherwise, KB1 may utilize one of the alternative pathways of glycine betaine catabolism. Yakimov et al. (2013) suggested that KB1 might produce both acetate and trimethylamine, and grow as a syntroph of methylotrophic archaea, following previous results that glycine betaine-metabolizing bacteria can form syntrophic associations with methylotrophic methanogens (e.g., King, 1984). Enrichments dominated by KB1 and MSBL1 16S rRNA sequences incorporated ^14^C-labeled glycine betaine and produced trimethylamine following glycine betaine addition (Yakimov et al., 2013). MSBL1 were recently proposed to be mixotrophic sugar-fermenting organisms based on single cell genomic analysis (Mwirichia et al., 2016). The cleavage enzyme (glycine betaine reductase) that traditionally produces acetate and trimethylamine was not detected in Orca Basin KB1, but could not be discounted due to the fragmentary SAG. Potentially, KB1 may utilize both demethylation and cleavage of glycine betaine, depending on environmental factors and potential syntrophic relationships.

### Phylogenetic Analysis of Acetyl-CoA Pathway Genes and Metabolic Function

**FIGURE 4:**
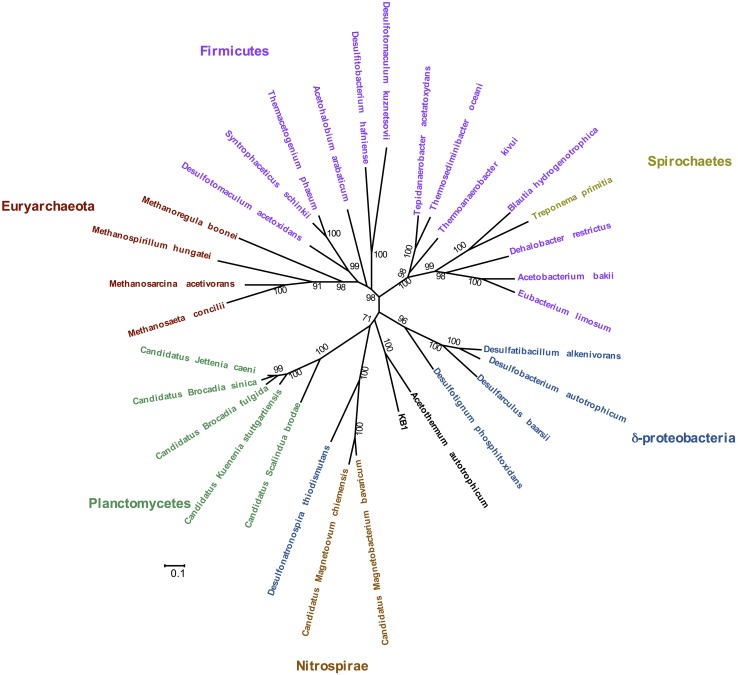
**Maximum Likelihood tree of concatenated CooS and AcsB protein sequences in the acetyl-CoA pathway, including the KB1 homolog [peg248] to *Candidatus* Acetothermum autotrophicum.** The Maximum Likelihood tree was constructed with a LG model with gamma distribution and invariant sites (LG+G+I) determined by using the model selection tool in MEGA. Amino acid alignment positions with less than 20% informative information (including gaps) were not considered. Scale bar indicates 0.1 amino acid substitutions per site. Bootstrap statistical support (>50%) based on 1000 replicates is displayed next to each node.

Phylogenetic analysis of the concatenated acetyl-CoA pathway proteins carbon monoxide dehydrogenase (subunit CooS) and the acetyl-CoA synthase (subunit AcsB) indicate monophyly of Orca Basin KB1 with *C.* Acetothermum autotrophicum (**Figure 4**). Complete enzymes of the acetyl-CoA pathway have been recognized in the genome of *C.* Acetothermum autotrophicum within the Acetothermi group (Takami et al., 2012) (**Figure 1**).

The acetyl-CoA pathway is known to operate in both directions (e.g., Hattori et al., 2005). In *C.* Acetothermum autotrophicum, it was proposed to function autotrophically by fixing CO_2_ with H_2_ (Takami et al., 2012). KB1 may also be able to utilize the pathway to fix CO_2_ when carbon is scarce. As a further possibility, KB1 may also produce acetyl-CoA, in combination with heterotrophy (Schuchmann and Müller, 2014). Genes encoding the protein subunits of pyruvate:ferredoxin oxidoreductase (EC 1.2.7.1) were also observed in the genome (RAST peg 635–637) indicating another possibility for production of acetyl-CoA utilizing the Wood–Ljungdahl pathway, potentially in combination with methyl groups derived from glycine betaine demethylation (Supplementary Figure S6) (Kreher et al., 2008). Several genes for acetyl-CoA fermentation to butyrate were also present, though not all genes comprising the pathway were observed in the partial KB1 genome (Supplementary Figure S8).

### Implications

The Candidate Division OP1/KB1 bacteria are deeply rooted on the tree of life (Takami et al., 2012). The four distinct groups of the OP1/KB1 complex (**Figure 1**) were consistent with class-level taxa according to the Greengenes taxonomic classification. Therefore, it is proposed to retain OP1, KB1, MSBL6, and the Acetothermia as four separate groups on class level which are combined into a single phylum; this conservative solution should be preferable to the alternative of elevating these class-level taxa to four different phyla within a superphylum, analogous to previously proposed cases (Wagner and Horn, 2006). The acidic amino acid bias indicated evolutionary change in KB1 from its common ancestor with OP1, allowing it to thrive under hypersaline conditions, and likely prohibiting it from living at low salt concentration. The acetyl-CoA pathway is potentially orthologous among KB1 and OP1, but glycine betaine uptake proteins and methyltransferases were possibly horizontally transferred. KB1 evolved a unique combination of protein modifications and compatible solute uptake and utilization pathways to survive in hypersaline environments.

## Author Contributions

LMN and AT designed the study. LMN and ASH performed the data analysis and constructed the figures. LMN and AT wrote the manuscript. BJM gave critical feedback and contributed to the text as well as the interpretation of results.

## Conflict of Interest Statement

The authors declare that the research was conducted in the absence of any commercial or financial relationships that could be construed as a potential conflict of interest.

## Footnotes

1 http://www.uniprot.org

2 http://web.expasy.org/compute_pi/
