# Supplementary material for: Phylogeography, Salinity Adaptations and Metabolic Potential of the Candidate Division KB1 Bacteria Based on a Partial Single Cell Genome
Source: Front Microbiol. 2016 Aug 22;7:1266. doi: 10.3389/fmicb.2016.01266 (PMC4993014; doi:10.3389/fmicb.2016.01266)
Supplement: Supplementary file 1 [file Data_Sheet_1.PDF]

frontiers

Supplementary Material

Phylogeography, salinity adaptations and metabolic potential of the Candidate Division KB1 Bacteria based on a partial single cell genome

Lisa M. Nigro*, Andrew S. Hyde, Barbara J. MacGregor and Andreas Teske

*** Correspondence:** Lisa M. Nigro: lnigro@uwf.edu

Supplementary Figures

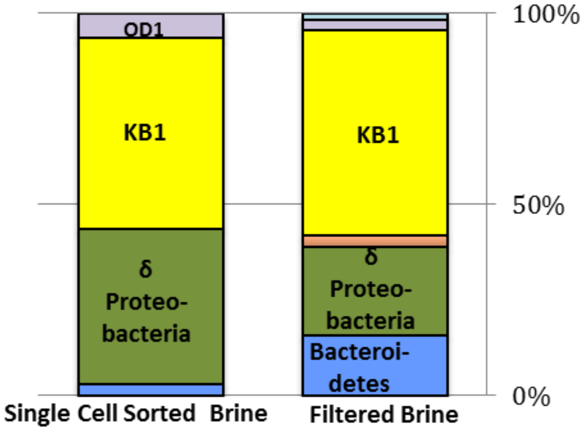

Figure S1. Percent taxonomic abundance of bacterial sequences amplified from single cell sorted Orca Basin brine and brine filtered through a 0.22 filter from a previous research cruise (R/V *Atlantis* leg AT18-22, Nov 2010, Chief Scientist S.B. Joye). Genbank Accession numbers associated with sequences and metadata: KX608684-KX608715; KR857575-KR857679.

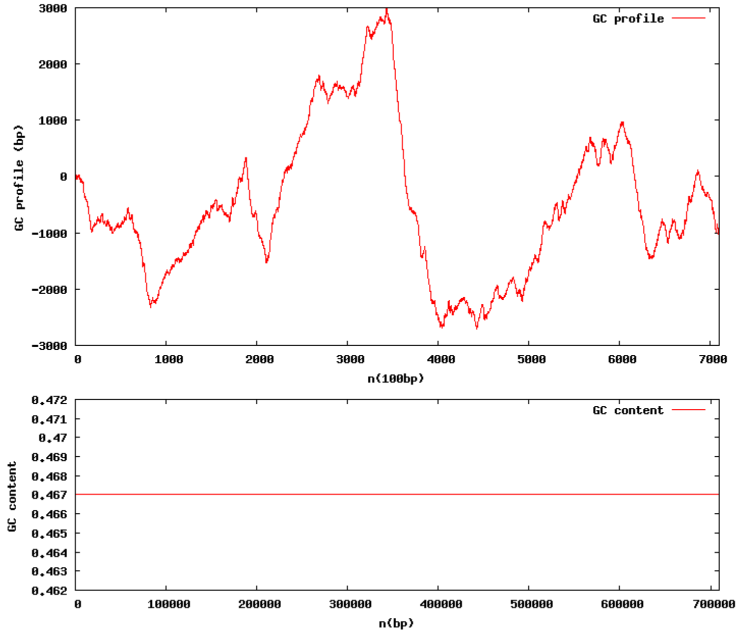

Figure S2. GC profile and % content calculated in GC-Profile (Gao and Zhang, 2006) with a minimum length of 1000 bp and a halting parameter of 50.

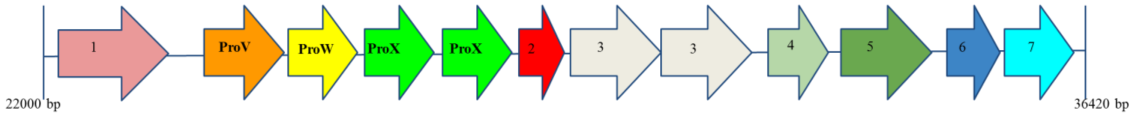

Figure S3. The KB1 genome contains a potential ProU ATP binding cassette operon (ProVWX) for glycine betaine transport into the cell. The ProU region is adjacent to putative protein encoding genes 1) putative methyltransferase 2) 5-methyl-THF:corrinoid methyltransferase, 3) Glycine decarboxylase (P1 and P2 proteins), 4) 5-methyltetrahydrofolate--homocysteine methyltransferase 5) formyl-THF-ligase 6) GTP cyclohydrolase I 7) methylene-THF dehydrogenase.

PROV

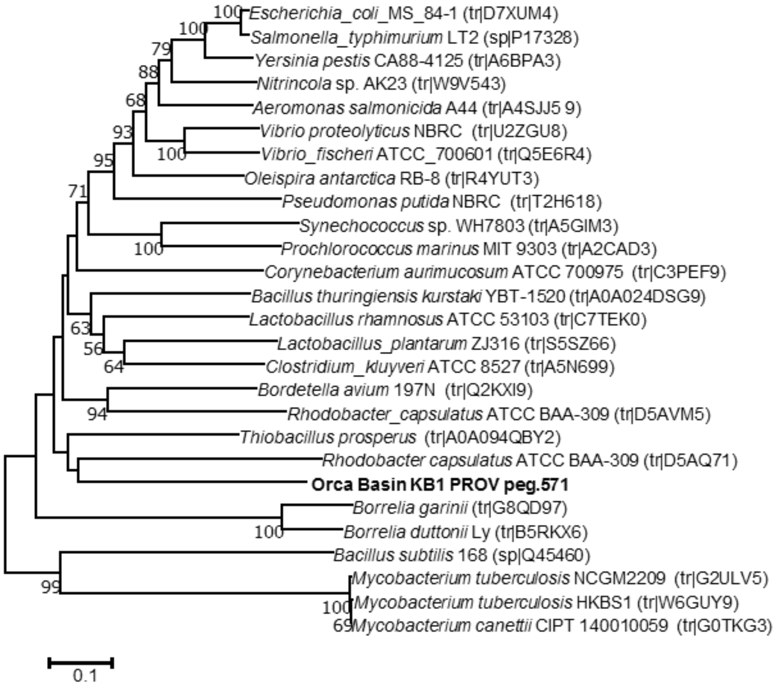
Escherichia_coli_MS_84-1 (tr|D7XUM4)
Salmonella_typhimurium LT2 (sp|P17328)
Yersinia pestis CA88-4125 (tr|A6BPA3)
Nitrincola sp. AK23 (tr|W9V543)
Aeromonas salmonicida A44 (tr|A4SJJ5 9)
Vibrio proteolyticus NBRC (tr|U2ZGU8)
Vibrio_fischeri ATCC_700601 (tr|Q5E6R4)
Oleispira antarctica RB-8 (tr|R4YUT3)
Pseudomonas putida NBRC (tr|T2H618)
Synechococcus sp. WH7803 (tr|A5GIM3)
Prochlorococcus marinus MIT 9303 (tr|A2CAD3)
Corynebacterium aurimucosum ATCC 700975 (tr|C3PEF9)
Bacillus thuringiensis kurstaki YBT-1520 (tr|A0A024DSG9)
Lactobacillus rhamnosus ATCC 53103 (tr|C7TEK0)
Lactobacillus_plantarum ZJ316 (tr|S5SZ66)
Clostridium_kluyveri ATCC 8527 (tr|A5N699)
Bordetella avium 197N (tr|Q2KXI9)
Rhodobacter_capsulatus ATCC BAA-309 (tr|D5AVM5)
Thiobacillus prosperus (tr|A0A094QBY2)
Rhodobacter capsulatus ATCC BAA-309 (tr|D5AQ71)
Orca Basin KB1 PROV peg.571
Borrelia garinii (tr|G8QD97)
Borrelia duttonii Ly (tr|B5RKX6)
Bacillus subtilis 168 (sp|Q45460)
Mycobacterium tuberculosis NCGM2209 (tr|G2ULV5)
Mycobacterium tuberculosis HKBS1 (tr|W6GUY9)
Mycobacterium canettii CIPT 140010059 (tr|G0TKG3)
0.1

PROW

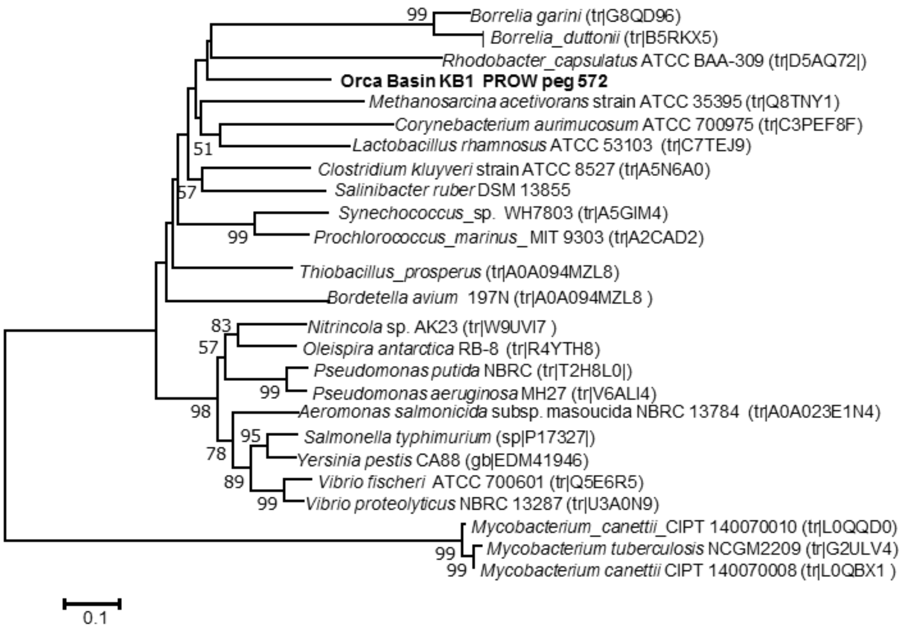
Borrelia garini (tr|G8QD96)
Borrelia_duttonii (tr|B5RKX5)
Rhodobacter_capsulatus ATCC BAA-309 (tr|D5AQ72|)
Orca Basin KB1 PROW peg 572
Methanosarcina acetivorans strain ATCC 35395 (tr|Q8TNY1)
Corynebacterium aurimucosum ATCC 700975 (tr|C3PEF8F)
Lactobacillus rhamnosus ATCC 53103 (tr|C7TEJ9)
Clostridium kluyveri strain ATCC 8527 (tr|A5N6A0)
Salinibacter ruber DSM 13855
Synechococcus_sp. WH7803 (tr|A5GIM4)
Prochlorococcus_marinus_ MIT 9303 (tr|A2CAD2)
Thiobacillus_prosperus (tr|A0A094MZL8)
Bordetella avium 197N (tr|A0A094MZL8)
Nitrincola sp. AK23 (tr|W9UVI7)
Oleispira antarctica RB-8 (tr|R4YTH8)
Pseudomonas putida NBRC (tr|T2H8L0|)
Pseudomonas aeruginosa MH27 (tr|V6ALI4)
Aeromonas salmonicida subsp. masoucida NBRC 13784 (tr|A0A023E1N4)
Salmonella typhimurium (sp|P17327|)
Yersinia pestis CA88 (gb|EDM41946)
Vibrio fischeri ATCC 700601 (tr|Q5E6R5)
Vibrio proteolyticus NBRC 13287 (tr|U3A0N9)
Mycobacterium_canettii_CIPT 140070010 (tr|L0QQD0)
Mycobacterium tuberculosis NCGM2209 (tr|G2ULV4)
Mycobacterium canettii CIPT 140070008 (tr|L0QBX1)
0.1

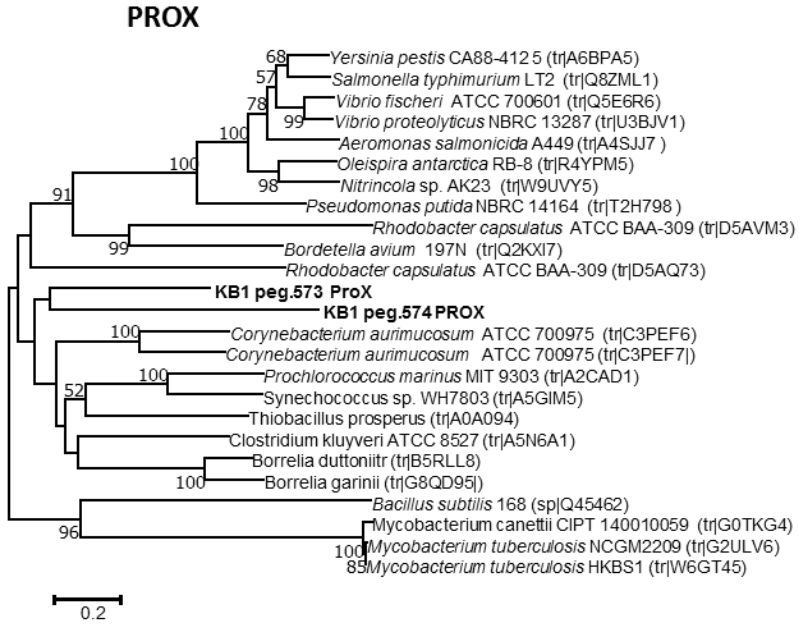

Figure S4. Neighbor Joining tree based on a Poisson model of the ProU operon proteins ProV, ProW, and ProX. KB1 putative proteins are in bold. Uninformative sites (including gaps) were not considered. Bootstrap statistical support (>50%) based on 1000 replicates is displayed next to each node.

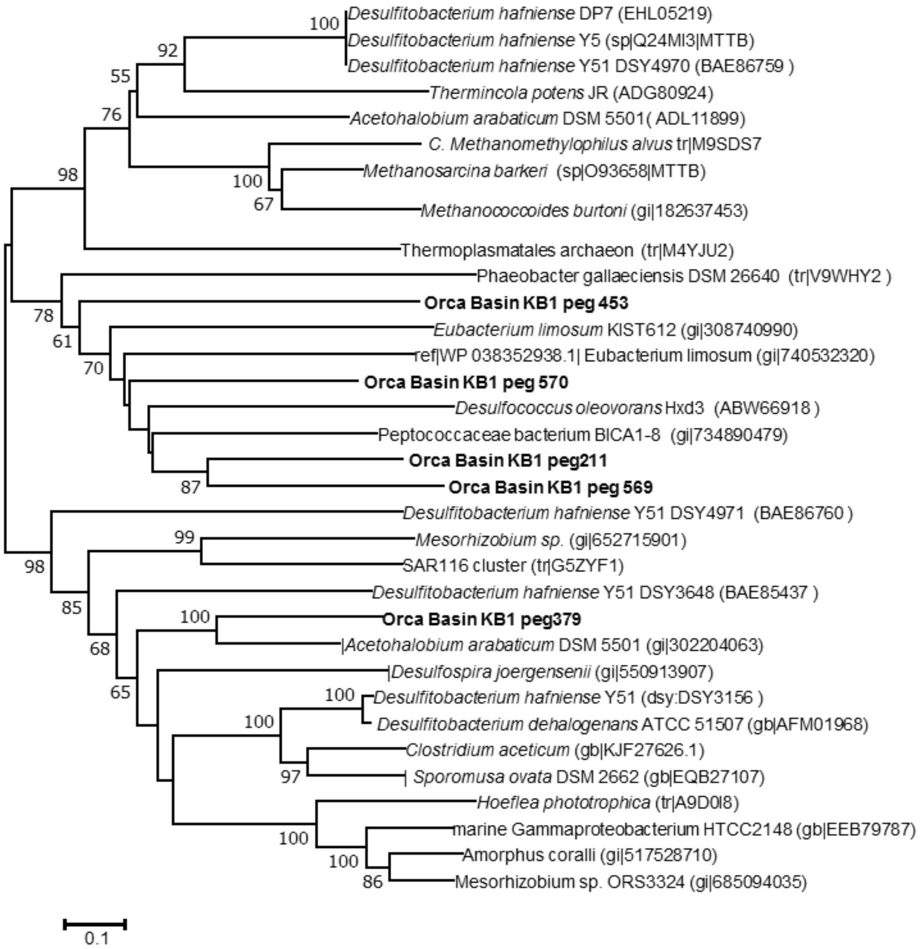

Figure S5. Neighbor Joining tree of putative corrinoid methyltransferases. KB1 sequences are in bold. The tree was constructed with a Poisson model and 1000 bootstrap replicates. Uninformative sites (including gaps) were not considered. Bootstrap statistical support (>50%) based on 1000 replicates is displayed next to each node.

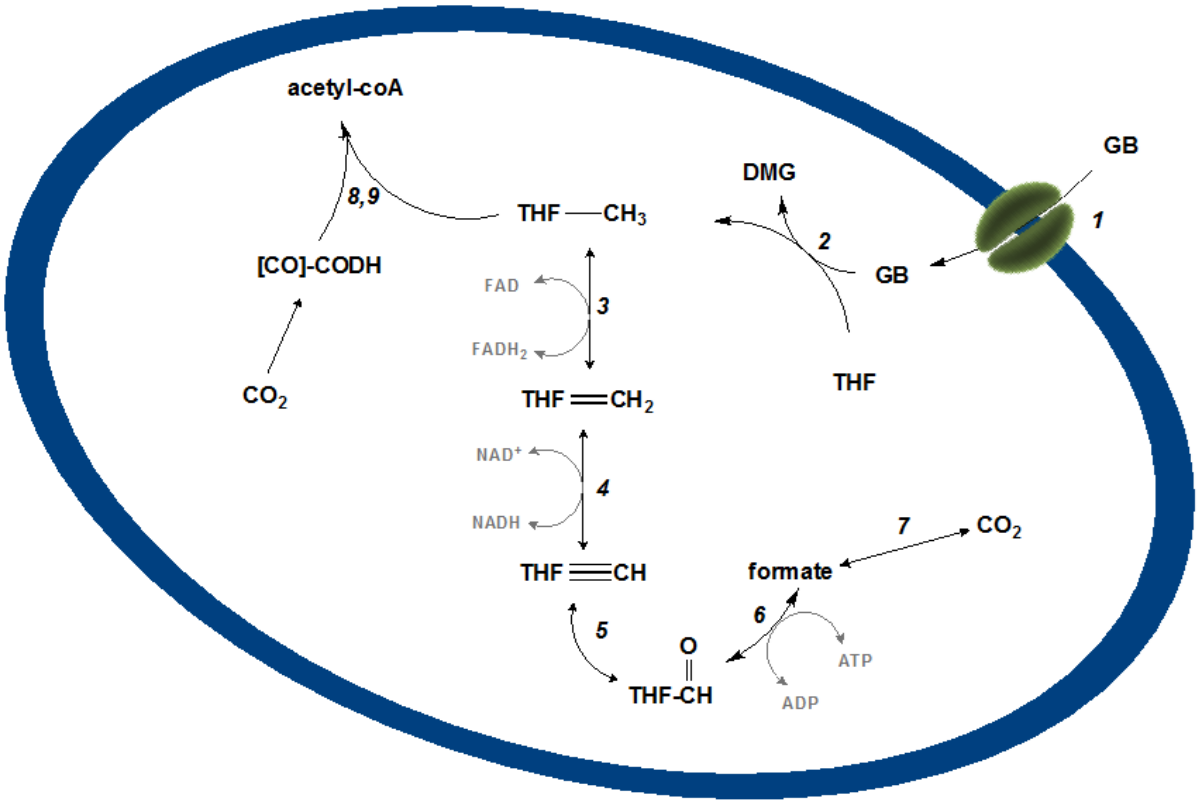

Figure S6. Possible fate of glycine betaine (GB) in KB1. Enzyme Commission number (EC) and protein encoding gene (peg) identification number in RAST are noted where available. Step 1, ATP binding cassette; T.C.3.A.1.12.1. Step 2, trimethylamine methyltransferase, peg 578. Step 3, methylene-THF reductase, E.C. 1.5.1.20, peg 555. Step 4, methylene-THF dehydrogenase, E.C.1.5.1.5, peg 581. Step 5, methenyl-THF-cyclohydrolase; E.C.3.5.4.9, peg 581. Step 6, formyl-THF-ligase, E.C.6.3.4.3, peg 579. Step 7, formate dehydrogenase, E.C. 1.2.1.43, peg 563, and EC1.2.1.2, peg 234. Step 8, 5-methyl-THF:corrinoid methyltransferase; E.C.2.1.1.258, peg 575. Step 9, complex of carbon monoxide dehydrogenase (CODH), E.C.1.2.99.2, peg 248, and acetyl-coA synthase (ACS), peg 243, 244, 246.

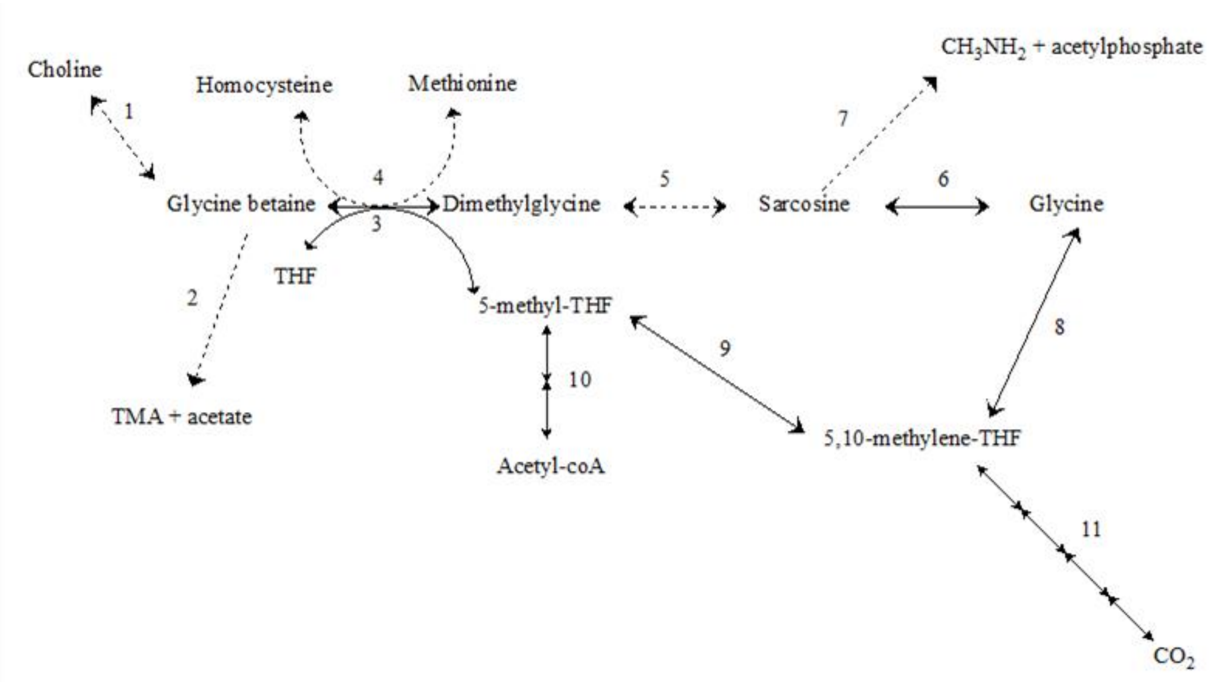

Figure S7. Potential metabolic fates of glycine betaine and its derivatives. 1) choline oxidase, E.C. 1.1.3.17; 2) glycine betaine reductase (E.C. 1.21.4.4); 3) Trimethylamine methyl-transferase 4) betaine-homocysteine S-methyltransferase, E.C.2.1.1.5; 5) dimethylglycine oxidase, E.C. 1.4.4.2; 6) sarcosine oxidase, E.C. 1.5.3.1; 7) sarcosine reductase, E.C. 1.21.4.3; 8) glycine dehydrogenase, E.C. 1.4.4.2; 9) Methylene-THF-reductase, E.C. 1.5.1.20; 10) subsequent reactions involving a corrinoid:methyltransferase and the carbon monoxide dehydrogenase/acetyl-CoA synthetase complex result in the formation of Acetyl-CoA; 11) the bidirectional conversion of CO_2 to acetyl-CoA (Wood-Ljungdahl pathway). Dotted pathways indicate enzymatic reactions not found in the KB1 Orca basin partial genome; solid lines are putatively present in the Orca Basin KB1 genome.

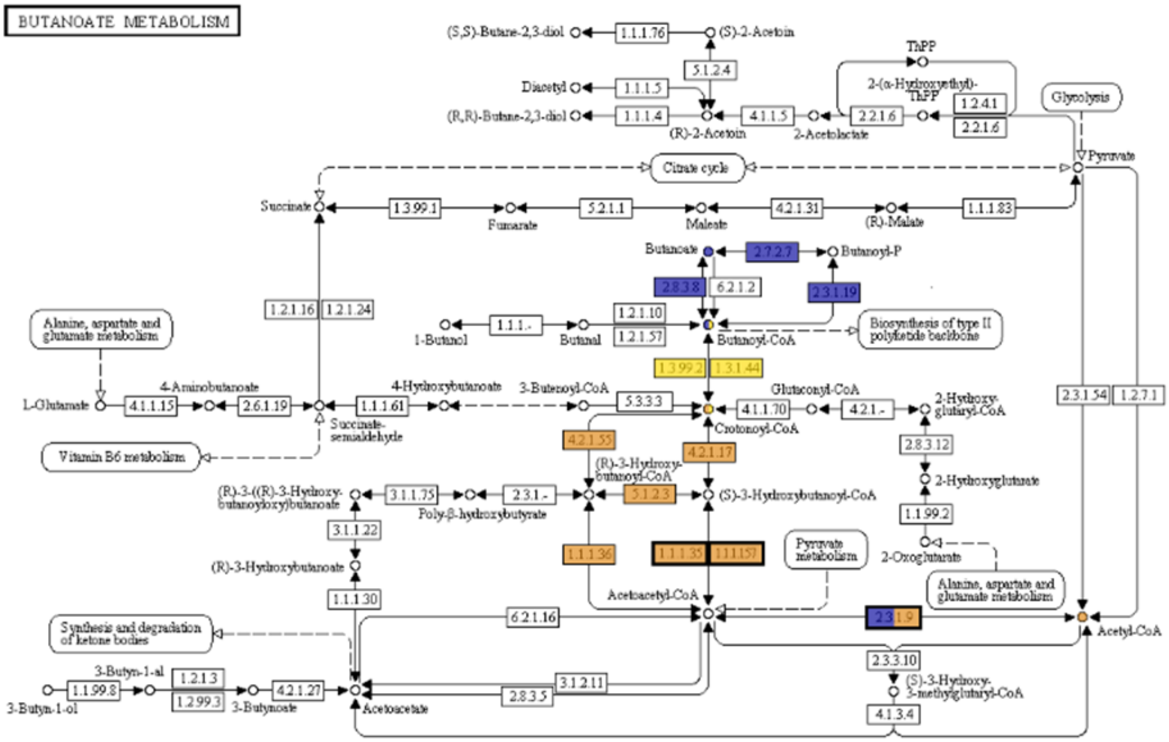

Figure S8: Potential fermentation of Acetyl CoA to butanol. Highlighted portions in pathways (Acetyl-CoA to Crotonyl CoA, orange; Crotonoyl-CoA to Butanoyl CoA, yellow; and butanoyl CoA to butanoate,purple) indicate possible presence in the partial KB1 genome. Numbers in boxes indicate enzyme commission (EC) classifications. Figure produced in RAST.

Supplemental Tables

Supplemental Table 1: Genome Completeness

Gene	Description	In KB1?	PEG
alaS	alanyl-tRNA synthetase		
argS	arginyl-tRNA synthetase		
aspS	aspartyl-tRNA synthetase		
cgtA	obg family GTPase CgtA	yes	230
coaE	dephospho-coA kinase		
cysS	cysteinyl-tRNA synthetase		
dnaA	chromosomal replication initiator protein DnaA		
dnaG	DNA primase		
dnaK	chaperone protein DnaK	yes	98
dnaN	DNA polymerase III, beta-subunit		
dnaX	DNA polyermase III, subunits gamma and tau	yes	438
engA	ribosome-associated GTPase EngA		
era	GTP=binding protein Era		
ffh	signal recognition particle protein		
fmt	methionyl-tRNA formyltransferase		
frr	ribosome recycling factor		
ftsY	signal recognition particle-docking protein FtsY		
glyS	glycyl-tRNA synthetase	yes	225
glyS	glycyl-tRNA synthetase	yes	224
gmk	guanylate kinase		
grpE	co-chaperone GrpE	yes	99
gyrA	DNA gyrase, A subunit	yes	448
gyrB	DNA gyrase, B subunit	yes	11
hisS	histidyl-tRNA synthetase	yes	357
ileS	isoleucyl-tRNA synthetase		
infB	translation initiation factor IF2		
infC	translation initiation factor IF3	yes	228
ksgA	dimethyladenosine transferase		
lepA	GTP-binding protein LepA		
leuS	leucyl-tRNA synthetase		
ligA	DNA ligase, NAD-dependent		
mnmA	tRNA (5-methylaminomethyl-2-thiouridylate)-methyltransferase	yes	266
mraW	MraW methylase family		
nusA	transciption termination factor NusA	yes	518&519
nusG	transcription termination/antitermination factor NusG		
pgk	phosphoglyerate kinase	yes	508
pheS	phenylalanyl-tRNA synthetase, alpha subunit		
pheT	phenylalanyl-tRNA synthetase, beta subunit		
pheT	phenylalanyl-tRNA synthetase, beta subunit		
prfA	peptide chaine release factor 1		
proS	prolyl-tRNA synthetase	yes	407
proS	prolyl-tRNA synthetase		
pyrG	CTP synthase		
recA	recA protein	yes	609
rfbA	ribosome-binding factor A		

rnc	ribonuclease III		
rplA	ribosome protein L1		
rplB	ribosome protein L2		
rplC	ribosome protein L3		
rplD	ribosome protein L4		
rplE	ribosome protein L5		
rplF	ribosome protein L6		
rplI	ribosome protein L9		
rplJ	ribosome protein L10		
rplK	ribosome protein L11		
rplL	ribosome protein L7/12		
rplM	ribosome protein L13		
rplN	ribosome protein L14		
rplO	ribosome protein L15		
rplP	ribosome protein L16		
rplQ	ribosome protein L17		
rplR	ribosome protein L18		
rplS	ribosome protein L19		
rplT	ribosome protein L20	yes	226
rplU	ribosome protein L21	yes	232
rplV	ribosome protein L22		
rplW	ribosome protein L23		
rplX	ribosome protein L24		
rpmA	ribosome protein L27	yes	231
rpmB	ribosome protein L28		
rpmC	ribosome protein L29		
rpmF	ribosome protein L32		
rpmH	ribosome protein L34		
rpmI	ribosome protein L35		
rpoA	DNA-directed RNA polymerase, alpha subunit		
rpoB	DNA-directed RNA polymerase, beta subunit		
rpoC	DNA-directed RNA polymerase, beta' and beta" subunit		
rpoC	DNA-directed RNA polymerase, beta' and beta" subunit		
rpsB	ribosomal protein S2		
rpsC	ribosomal protein S3		
rpsD	ribosomal protein S4		
rpsE	ribosomal protein S5		
rpsF	ribosomal protein S6	yes	440
rpsG	ribosomal protein S7		
rpsH	ribosomal protein S8		
rpsI	ribosomal protein S9		
rpsJ	ribosomal protein S10		
rpsK	ribosomal protein S11		
rpsL	ribosomal protein S12		
rpsM	ribosomal protein S13		
rpsO	ribosomal protein S15		
rpsP	ribosomal protein S16		
rpsQ	ribosomal protein S17		
rpsR	ribosomal protein S18	yes	439
rpsS	ribosomal protein S19		
rpsT	ribosomal protein S20		
secA	preprotein translocase, secA subunit	yes	598

secE	preprotein translocase, secE subunit		
secG	preprotein translocase, secG subunit		
secY	preprotein translocase, secY subunit		
serS	seryl-tRNA synthetase		
smpB	smpB protein		
thrS	threonyl-tRNA synthetase	yes	377
tig	trigger factor	yes	121
tilS	tRNA(Ile)-lysidine synthase	yes	369
tsf	translation elongation factor Ts		
tyrS	tyrosyl-tRNA synthetase	yes	449
uvrB	excinuclease ABC, B subunit	yes	303
valS	valyl-tRNA synthetase	yes	341
ybeY	conserved hypothetical protein, YbeY		
ychF	GTP-binding protein YchF		
	Number of selected genes present in KB1	27	
	Genome completeness estimate	0.25	

Supplemental Table 2: Identified Subsystem Genes in RAST

Vitamins and Cofactors

**Subsystem**	**Role**
Biotin biosynthesis	Long-chain-fatty-acid--CoA ligase (EC 6.2.1.3)
Biotin biosynthesis	3-ketoacyl-CoA thiolase (EC 2.3.1.16)
Biotin biosynthesis	Substrate-specific component BioY of biotin ECF transporter
Cobalamin synthesis	L-threonine 3-O-phosphate decarboxylase (EC 4.1.1.81)
Cobalamin synthesis	Adenosylcobinamide-phosphate guanylyltransferase (EC 2.7.7.62)
Cobalamin synthesis	Cobalamin synthase (EC 2.7.8.26)
Cobalamin synthesis	Nicotinate-nucleotide--dimethylbenzimidazole phosphoribosyltransferase (EC 2.4.2.21)
Cobalamin synthesis	Adenosylcobinamide-phosphate synthase (EC 6.3.1.10)
riboflavin to FAD	FMN adenylyltransferase (EC 2.7.7.2)
riboflavin to FAD	Riboflavin kinase (EC 2.7.1.26)
Flavodoxin	Flavodoxin
Riboflavin, FMN and FAD metabolism	FMN adenylyltransferase (EC 2.7.7.2)
Riboflavin, FMN and FAD metabolism	Diaminohydroxyphosphoribosylaminopyrimidine deaminase (EC 3.5.4.26)
Riboflavin, FMN and FAD metabolism	5-amino-6-(5-phosphoribosylamino)uracil reductase (EC 1.1.1.193)
Riboflavin, FMN and FAD metabolism	Riboflavin kinase (EC 2.7.1.26)
NAD and NADP cofactor biosynthesis global	Nicotinate phosphoribosyltransferase (EC 2.4.2.11)
NAD and NADP cofactor biosynthesis global	NAD kinase (EC 2.7.1.23)
NAD and NADP cofactor biosynthesis global	Nicotinate-nucleotide adenylyltransferase (EC 2.7.7.18)
Molybdenum cofactor biosynthesis	Molybdenum cofactor biosynthesis protein MoaC
Molybdenum cofactor biosynthesis	Molybdopterin biosynthesis protein MoeA
Molybdenum cofactor biosynthesis	Molybdopterin-guanine dinucleotide biosynthesis protein MobA
Molybdenum cofactor biosynthesis	GTP cyclohydrolase I (EC 3.5.4.16) type 1
Molybdenum cofactor biosynthesis	Molybdenum cofactor biosynthesis protein MoaA
Molybdenum cofactor biosynthesis	FIG060329: MOSC domain protein
Molybdenum cofactor biosynthesis	Molybdenum cofactor biosynthesis protein MoaB

Molybdenum cofactor biosynthesis	Periplasmic molybdate-binding domain
Molybdenum cofactor biosynthesis	Molybdopterin biosynthesis protein MoeB
Folate Biosynthesis	Folylpolyglutamate synthase (EC 6.3.2.17)
Folate Biosynthesis	Dihydropteroate synthase (EC 2.5.1.15)
Folate Biosynthesis	FolM Alternative dihydrofolate reductase 1
Folate Biosynthesis	Thymidylate synthase thyX (EC 2.1.1.-)
Folate Biosynthesis	Dihydrofolate synthase (EC 6.3.2.12)
Folate Biosynthesis	GTP cyclohydrolase I (EC 3.5.4.16) type 1
Folate Biosynthesis	Dihydroneopterin aldolase (EC 4.1.2.25)
Folate Biosynthesis	Para-aminobenzoate synthase, amidotransferase component (EC 2.6.1.85)
Pterin carbinolamine dehydratase	Pterin-4-alpha-carbinolamine dehydratase (EC 4.2.1.96)

Cell Wall

UDP-N-acetylmuramate from Fructose-6-phosphate Biosynthesis	Glucosamine-1-phosphate N-acetyltransferase (EC 2.3.1.157)
UDP-N-acetylmuramate from Fructose-6-phosphate Biosynthesis	Glucosamine--fructose-6-phosphate aminotransferase [isomerizing] (EC 2.6.1.16)
UDP-N-acetylmuramate from Fructose-6-phosphate Biosynthesis	UDP-N-acetylglucosamine 1-carboxyvinyltransferase (EC 2.5.1.7)
UDP-N-acetylmuramate from Fructose-6-phosphate Biosynthesis	N-acetylglucosamine-1-phosphate uridyltransferase (EC 2.7.7.23)
Recycling of Peptidoglycan Amino Acids	Aminoacyl-histidine dipeptidase (Peptidase D) (EC 3.4.13.3)

Metal/ion transport, metabolism, homeostasis

Copper homeostasis	Copper-translocating P-type ATPase (EC 3.6.3.4)
Potassium homeostasis	Potassium-transporting ATPase A chain (EC 3.6.3.12) (TC 3.A.3.7.1)
Potassium homeostasis	Trk system potassium uptake protein TrkA
Potassium homeostasis	Potassium-transporting ATPase B chain (EC 3.6.3.12) (TC 3.A.3.7.1)
Potassium homeostasis	Potassium-transporting ATPase C chain (EC 3.6.3.12) (TC 3.A.3.7.1)
Magnesium transport	Mg/Co/Ni transporter MgtE
Copper Transport System	Copper-translocating P-type ATPase (EC 3.6.3.4)
Ferrous iron transporter EfeUOB, low-pH-induced	Ferrous iron transport permease EfeU

Amino acid membrane transport

ABC transporter branched-chain amino acid (TC 3.A.1.4.1)	High-affinity branched-chain amino acid transport system permease protein LivH (TC 3.A.1.4.1)
ABC transporter branched-chain amino acid (TC 3.A.1.4.1)	Branched-chain amino acid transport ATP-binding protein LivF (TC 3.A.1.4.1)
ABC transporter branched-chain amino acid (TC 3.A.1.4.1)	Branched-chain amino acid transport system permease protein LivM (TC 3.A.1.4.1)
ABC transporter branched-chain amino acid (TC 3.A.1.4.1)	Branched-chain amino acid ABC transporter, amino acid-binding protein (TC 3.A.1.4.1)
ABC transporter branched-chain amino acid (TC 3.A.1.4.1)	Branched-chain amino acid transport ATP-binding protein LivG (TC 3.A.1.4.1)

RNA Metabolism

tRNA nucleotidyltransferase	tRNA nucleotidyltransferase (EC 2.7.7.21) (EC 2.7.7.25)
Methylthiotransferases	tRNA-i(6)A37 methylthiotransferase
mnm5U34 biosynthesis bacteria	tRNA (5-methylaminomethyl-2-thiouridylate)-methyltransferase (EC 2.1.1.61)
mnm5U34 biosynthesis bacteria	Cysteine desulfurase (EC 2.8.1.7)
Transcription factors bacterial	Transcription termination protein NusB
Transcription factors bacterial	Transcription termination factor Rho
Transcription factors bacterial	FIG000325: clustered with transcription termination protein NusA
Transcription factors bacterial	Transcription termination protein NusA

Nucleoside and Nucleotide related

pyrimidine conversions	Bis(5'-nucleosyl)-tetraphosphatase (asymmetrical) (EC 3.6.1.17)
pyrimidine conversions	Purine nucleoside phosphorylase (EC 2.4.2.1)
pyrimidine conversions	Nucleoside diphosphate kinase (EC 2.7.4.6)
pyrimidine conversions	Thioredoxin reductase (EC 1.8.1.9)
pyrimidine conversions	Pyrimidine-nucleoside phosphorylase (EC 2.4.2.2)
pyrimidine conversions	Deoxycytidine triphosphate deaminase (EC 3.5.4.13)
GMP synthase	GMP synthase [glutamine-hydrolyzing], ATP pyrophosphatase subunit (EC 6.3.5.2)
GMP synthase	GMP synthase [glutamine-hydrolyzing], amidotransferase subunit (EC 6.3.5.2)
Hydantoin metabolism	N-methylhydantoinase (ATP-hydrolyzing) (EC 3.5.2.14)
Adenosyl nucleosidases	Purine nucleoside phosphorylase (EC 2.4.2.1)

Protein Metabolism

Protein chaperones	Heat shock protein GrpE
Protein chaperones	Chaperone protein DnaK
Protein chaperones	Chaperone protein DnaJ
Protein chaperones	ClpB protein
Peptidyl-prolyl cis-trans isomerase	Foldase protein PrsA precursor (EC 5.2.1.8)
tRNA aminoacylation, Val	Valyl-tRNA synthetase (EC 6.1.1.9)
tRNA aminoacylation, Met	Methionyl-tRNA synthetase (EC 6.1.1.10)
tRNA aminoacylation, His	Histidyl-tRNA synthetase (EC 6.1.1.21)
tRNA aminoacylation, Asp and Asn	Aspartyl-tRNA synthetase (EC 6.1.1.12)
tRNA aminoacylation, Asp and Asn	Asparaginyl-tRNA synthetase (EC 6.1.1.22)
tRNA aminoacylation, Asp and Asn	Aspartyl-tRNA(Asn) amidotransferase subunit A (EC 6.3.5.6)
tRNA aminoacylation, Gly	Glycyl-tRNA synthetase alpha chain (EC 6.1.1.14)
tRNA aminoacylation, Gly	Glycyl-tRNA synthetase beta chain (EC 6.1.1.14)
tRNA aminoacylation, Thr	Threonyl-tRNA synthetase (EC 6.1.1.3)
tRNA aminoacylation, Glu and Gln	Glutamyl-tRNA(Gln) amidotransferase subunit A (EC 6.3.5.7)
tRNA aminoacylation, Glu and Gln	Glutamyl-tRNA synthetase (EC 6.1.1.17)
tRNA aminoacylation, Glu and Gln	Glutamyl-tRNA(Gln) synthetase (EC 6.1.1.24)
tRNA aminoacylation, Tyr	Tyrosyl-tRNA synthetase (EC 6.1.1.1)
Glycyl-tRNA synthetase	Glycyl-tRNA synthetase alpha chain (EC 6.1.1.14)
Glycyl-tRNA synthetase	Glycyl-tRNA synthetase beta chain (EC 6.1.1.14)
Protein-L-isoaspartate O-methyltransferase	Protein-L-isoaspartate O-methyltransferase (EC 2.1.1.77)
Proteasome bacterial	ATP-dependent hsl protease ATP-binding subunit HslU
Proteasome bacterial	ATP-dependent Clp protease proteolytic subunit (EC 3.4.21.92)
Proteasome bacterial	ATP-dependent protease HslV (EC 3.4.25.-)
Dipeptidases (EC 3.4.13.-)	Aminoacyl-histidine dipeptidase (Peptidase D) (EC 3.4.13.3)
Proteolysis in bacteria, ATP-dependent	ATP-dependent hsl protease ATP-binding subunit HslU
Proteolysis in bacteria, ATP-dependent	ATP-dependent Clp protease proteolytic subunit (EC 3.4.21.92)
Proteolysis in bacteria, ATP-dependent	ClpB protein
Proteolysis in bacteria, ATP-dependent	ATP-dependent protease HslV (EC 3.4.25.-)
Omega peptidases (EC 3.4.19.-)	Acylamino-acid-releasing enzyme (EC 3.4.19.1)

Cell Division and Cell Cycle

Bacterial Cytoskeleton	Sporulation initiation inhibitor protein Soj
Bacterial Cytoskeleton	Chromosome (plasmid) partitioning protein ParB
Bacterial Cytoskeleton	Stage 0 sporulation protein J
Bacterial Cytoskeleton	Chromosome (plasmid) partitioning protein ParA

DNA Metabolism/Repair

RecA and RecX	RecA protein
RecA and RecX	Regulatory protein RecX

DNA repair system including RecA, MutS and a hypothetical protein	RecA protein
DNA repair system including RecA, MutS and a hypothetical protein	Regulatory protein RecX
Restriction-Modification System	Type III restriction-modification system methylation subunit (EC 2.1.1.72)
Restriction-Modification System	Type I restriction-modification system, restriction subunit R (EC 3.1.21.3)
DNA structural proteins, bacterial	Chromosome partition protein smc
DNA structural proteins, bacterial	DNA-binding protein HU
DNA structural proteins, bacterial	DNA-binding protein HBsu
DNA topoisomerases, Type II, ATP-dependent	DNA gyrase subunit B (EC 5.99.1.3)
DNA topoisomerases, Type II, ATP-dependent	DNA gyrase subunit A (EC 5.99.1.3)
DNA processing cluster	DNA polymerase III subunits gamma and tau (EC 2.7.7.7)

Fatty Acids, Lipids, and Isoprenoids

Fatty acid metabolism cluster	Long-chain-fatty-acid--CoA ligase (EC 6.2.1.3)
Fatty acid metabolism cluster	3-ketoacyl-CoA thiolase (EC 2.3.1.16)
Fatty acid metabolism cluster	3-hydroxyacyl-CoA dehydrogenase (EC 1.1.1.35)
Polyhydroxybutyrate metabolism	3-ketoacyl-CoA thiolase (EC 2.3.1.16)
Polyhydroxybutyrate metabolism	3-hydroxybutyryl-CoA dehydrogenase (EC 1.1.1.157)
Polyhydroxybutyrate metabolism	3-hydroxyacyl-CoA dehydrogenase (EC 1.1.1.35)
Polyhydroxybutyrate metabolism	Acetyl-CoA acetyltransferase (EC 2.3.1.9)
Hopanes	Squalene--hopene cyclase (EC 5.4.99.17)

Respiration

Carbon monoxide induced hydrogenase	5-methyltetrahydrofolate:corrinoid iron-sulfur protein methyltransferase
Carbon monoxide induced hydrogenase	Carbon monoxide dehydrogenase CooS subunit (EC 1.2.99.2)
Carbon monoxide induced hydrogenase	Acetyl-CoA synthase corrinoid activation protein
Carbon monoxide induced hydrogenase	CO dehydrogenase/acetyl-CoA synthase, acetyl-CoA synthase subunit (EC 2.3.1.169)
Carbon monoxide induced hydrogenase	CO dehydrogenase accessory protein CooC (nickel insertion)
Carbon monoxide induced hydrogenase	Acetyl-CoA synthase corrinoid iron-sulfur protein, large subunit
Carbon monoxide induced hydrogenase	Acetyl-CoA synthase corrinoid iron-sulfur protein, small subunit
Formate hydrogenase	Formate dehydrogenase chain D (EC 1.2.1.2)
Formate hydrogenase	NAD-dependent formate dehydrogenase alpha subunit

Compatible Solute uptake

Choline and Betaine Uptake and Betaine Biosynthesis	L-proline glycine betaine ABC transport system permease protein ProV (TC 3.A.1.12.1)
Choline and Betaine Uptake and Betaine Biosynthesis	Sarcosine oxidase alpha subunit (EC 1.5.3.1)
Choline and Betaine Uptake and Betaine Biosynthesis	Sarcosine oxidase beta subunit (EC 1.5.3.1)
Choline and Betaine Uptake and Betaine Biosynthesis	L-proline glycine betaine binding ABC transporter protein ProX (TC 3.A.1.12.1)
Choline and Betaine Uptake and Betaine Biosynthesis	L-proline glycine betaine ABC transport system permease protein ProW (TC 3.A.1.12.1)

Stress Response

Oxidative stress	Rubrerythrin
Oxidative stress	Ferric uptake regulation protein
Oxidative stress	transcriptional regulator, Crp/Fnr family
Oxidative stress	Superoxide reductase (EC 1.15.1.2)
Oxidative stress	Fe2+/Zn2+ uptake regulation proteins
Oxidative stress	Peroxide stress regulator
Glutathione: Biosynthesis and gamma-	Glutamate--cysteine ligase (EC 6.3.2.2)

glutamyl cycle	
Rubrerythrin	Rubrerythrin
Rubrerythrin	Rubredoxin
Rubrerythrin	Superoxide reductase (EC 1.15.1.2)
Cold shock, CspA family of proteins	Cold shock protein CspA
Hfl operon	GTP-binding protein HflX

Amino Acids and Derivatives

Glutamine, Glutamate, Aspartate and Asparagine Biosynthesis	Aspartate aminotransferase (EC 2.6.1.1)
Glutamine, Glutamate, Aspartate and Asparagine Biosynthesis	NADP-specific glutamate dehydrogenase (EC 1.4.1.4)
Glutamine, Glutamate, Aspartate and Asparagine Biosynthesis	NAD-specific glutamate dehydrogenase (EC 1.4.1.2)
Glutamate dehydrogenases	NADP-specific glutamate dehydrogenase (EC 1.4.1.4)
Glutamate dehydrogenases	NAD-specific glutamate dehydrogenase (EC 1.4.1.2)
Histidine Biosynthesis	Phosphoribosylformimino-5-aminoimidazole carboxamide ribotide isomerase (EC 5.3.1.16)
Histidine Biosynthesis	Phosphoribosyl-ATP pyrophosphatase (EC 3.6.1.31)
Histidine Biosynthesis	Imidazole glycerol phosphate synthase amidotransferase subunit (EC 2.4.2.-)
Histidine Biosynthesis	Histidinol-phosphate aminotransferase (EC 2.6.1.9)
Histidine Biosynthesis	Imidazoleglycerol-phosphate dehydratase (EC 4.2.1.19)
Histidine Biosynthesis	ATP phosphoribosyltransferase regulatory subunit (EC 2.4.2.17)
Histidine Biosynthesis	Imidazole glycerol phosphate synthase cyclase subunit (EC 4.1.3.-)
Histidine Biosynthesis	Phosphoribosyl-AMP cyclohydrolase (EC 3.5.4.19)
Histidine Biosynthesis	ATP phosphoribosyltransferase (EC 2.4.2.17)
Histidine Biosynthesis	Histidinol dehydrogenase (EC 1.1.1.23)
Threonine and Homoserine Biosynthesis	Homoserine dehydrogenase (EC 1.1.1.3)
Threonine and Homoserine Biosynthesis	Aspartate-semialdehyde dehydrogenase (EC 1.2.1.11)
Threonine and Homoserine Biosynthesis	Aspartate aminotransferase (EC 2.6.1.1)
Threonine and Homoserine Biosynthesis	Threonine synthase (EC 4.2.3.1)
Threonine and Homoserine Biosynthesis	Aspartokinase (EC 2.7.2.4)
Threonine and Homoserine Biosynthesis	Predicted functional analog of homoserine kinase (EC 2.7.1.-)
Threonine degradation	L-threonine 3-dehydrogenase (EC 1.1.1.103)
Creatine and Creatinine Degradation	N-methylhydantoinase (ATP-hydrolyzing) (EC 3.5.2.14)
Creatine and Creatinine Degradation	Creatinine amidohydrolase (EC 3.5.2.10)
Common Pathway For Synthesis of Aromatic Compounds (DAHP synthase to chorismate)	Shikimate kinase I (EC 2.7.1.71)
Common Pathway For Synthesis of Aromatic Compounds (DAHP synthase to chorismate)	5-Enolpyruvylshikimate-3-phosphate synthase (EC 2.5.1.19)
Common Pathway For Synthesis of Aromatic Compounds (DAHP synthase to chorismate)	Shikimate 5-dehydrogenase I alpha (EC 1.1.1.25)
Common Pathway For Synthesis of Aromatic Compounds (DAHP synthase to chorismate)	3-dehydroquinate dehydratase II (EC 4.2.1.10)
Common Pathway For Synthesis of Aromatic Compounds (DAHP synthase to chorismate)	2-keto-3-deoxy-D-arabino-heptulosonate-7-phosphate synthase I beta (EC 2.5.1.54)
Common Pathway For Synthesis of Aromatic Compounds (DAHP synthase to chorismate)	3-dehydroquinate synthase (EC 4.2.3.4)
Common Pathway For Synthesis of Aromatic Compounds (DAHP synthase to chorismate)	Chorismate synthase (EC 4.2.3.5)
Chorismate Synthesis	5-Enolpyruvylshikimate-3-phosphate synthase (EC 2.5.1.19)
Chorismate Synthesis	Chorismate mutase I (EC 5.4.99.5)
Chorismate Synthesis	3-dehydroquinate dehydratase II (EC 4.2.1.10)
Chorismate Synthesis	Prephenate dehydratase (EC 4.2.1.51)
Chorismate Synthesis	3-dehydroquinate synthase (EC 4.2.3.4)

Chorismate Synthesis	Prephenate and/or arogenate dehydrogenase (unknown specificity) (EC 1.3.1.12)(EC 1.3.1.43)
Chorismate Synthesis	Chorismate synthase (EC 4.2.3.5)
Chorismate Synthesis	Shikimate kinase I (EC 2.7.1.71)
Chorismate Synthesis	2-keto-3-deoxy-D-arabino-heptulosonate-7-phosphate synthase I beta (EC 2.5.1.54)
Chorismate Synthesis	Shikimate 5-dehydrogenase I alpha (EC 1.1.1.25)
Chorismate: Intermediate for synthesis of Tryptophan, PAPA antibiotics, PABA, 3-hydroxyanthranilate and more.	Phosphoribosylformimino-5-aminoimidazole carboxamide ribotide isomerase (EC 5.3.1.16)
Chorismate: Intermediate for synthesis of Tryptophan, PAPA antibiotics, PABA, 3-hydroxyanthranilate and more.	Anthranilate synthase, amidotransferase component (EC 4.1.3.27)
Chorismate: Intermediate for synthesis of Tryptophan, PAPA antibiotics, PABA, 3-hydroxyanthranilate and more.	Tryptophan synthase alpha chain (EC 4.2.1.20)
Chorismate: Intermediate for synthesis of Tryptophan, PAPA antibiotics, PABA, 3-hydroxyanthranilate and more.	Anthranilate phosphoribosyltransferase (EC 2.4.2.18)
Chorismate: Intermediate for synthesis of Tryptophan, PAPA antibiotics, PABA, 3-hydroxyanthranilate and more.	Tryptophan synthase beta chain (EC 4.2.1.20)
Chorismate: Intermediate for synthesis of Tryptophan, PAPA antibiotics, PABA, 3-hydroxyanthranilate and more.	Indole-3-glycerol phosphate synthase (EC 4.1.1.48)
Chorismate: Intermediate for synthesis of Tryptophan, PAPA antibiotics, PABA, 3-hydroxyanthranilate and more.	Anthranilate synthase, aminase component (EC 4.1.3.27)
Chorismate: Intermediate for synthesis of Tryptophan, PAPA antibiotics, PABA, 3-hydroxyanthranilate and more.	Phosphoribosylanthranilate isomerase (EC 5.3.1.24)
Chorismate: Intermediate for synthesis of Tryptophan, PAPA antibiotics, PABA, 3-hydroxyanthranilate and more.	Tryptophan synthase beta chain like (EC 4.2.1.20)
Chorismate: Intermediate for synthesis of Tryptophan, PAPA antibiotics, PABA, 3-hydroxyanthranilate and more.	Para-aminobenzoate synthase, amidotransferase component (EC 2.6.1.85)
Phenylalanine and Tyrosine Branches from Chorismate	Chorismate mutase I (EC 5.4.99.5)
Phenylalanine and Tyrosine Branches from Chorismate	Prephenate dehydratase (EC 4.2.1.51)
Phenylalanine and Tyrosine Branches from Chorismate	Prephenate and/or arogenate dehydrogenase (unknown specificity) (EC 1.3.1.12)(EC 1.3.1.43)
Tryptophan synthesis	Anthranilate synthase, amidotransferase component (EC 4.1.3.27)
Tryptophan synthesis	Tryptophan synthase alpha chain (EC 4.2.1.20)
Tryptophan synthesis	Anthranilate phosphoribosyltransferase (EC 2.4.2.18)
Tryptophan synthesis	Tryptophan synthase beta chain (EC 4.2.1.20)
Tryptophan synthesis	Indole-3-glycerol phosphate synthase (EC 4.1.1.48)
Tryptophan synthesis	Para-aminobenzoate synthase, amidotransferase component (EC 2.6.1.85)
Tryptophan synthesis	Anthranilate synthase, aminase component (EC 4.1.3.27)
Tryptophan synthesis	Phosphoribosylanthranilate isomerase (EC 5.3.1.24)
Glycine Biosynthesis	Serine hydroxymethyltransferase (EC 2.1.2.1)
Glycine Biosynthesis	L-threonine 3-dehydrogenase (EC 1.1.1.103)
Alanine biosynthesis	Cysteine desulfurase (EC 2.8.1.7)
Serine Biosynthesis	D-3-phosphoglycerate dehydrogenase (EC 1.1.1.95)
Serine Biosynthesis	Serine hydroxymethyltransferase (EC 2.1.2.1)

Carbohydrates

Pyruvate:ferredoxin oxidoreductase	Pyruvate:ferredoxin oxidoreductase, delta subunit (EC 1.2.7.1)
Pyruvate:ferredoxin oxidoreductase	Pyruvate:ferredoxin oxidoreductase, alpha subunit (EC 1.2.7.1)
Pyruvate:ferredoxin oxidoreductase	Pyruvate:ferredoxin oxidoreductase, beta subunit (EC 1.2.7.1)
Pyruvate:ferredoxin oxidoreductase	Pyruvate:ferredoxin oxidoreductase, gamma subunit (EC 1.2.7.1)
One-carbon metabolism by tetrahydropterines	Formate--tetrahydrofolate ligase (EC 6.3.4.3)
One-carbon metabolism by tetrahydropterines	Formiminotetrahydrofolate cyclodeaminase (EC 4.3.1.4)
One-carbon metabolism by tetrahydropterines	Methylenetetrahydrofolate dehydrogenase (NADP+) (EC 1.5.1.5)
One-carbon metabolism by tetrahydropterines	5,10-methylenetetrahydrofolate reductase (EC 1.5.1.20)
One-carbon metabolism by tetrahydropterines	Methenyltetrahydrofolate cyclohydrolase (EC 3.5.4.9)
Acetolactate synthase subunits	Acetolactate synthase large subunit (EC 2.2.1.6)
Acetolactate synthase subunits	Acetolactate synthase small subunit (EC 2.2.1.6)
Acetyl-CoA fermentation to Butyrate	Electron transfer flavoprotein, beta subunit
Acetyl-CoA fermentation to Butyrate	Electron transfer flavoprotein, alpha subunit
Acetyl-CoA fermentation to Butyrate	3-hydroxybutyryl-CoA dehydrogenase (EC 1.1.1.157)
Acetyl-CoA fermentation to Butyrate	3-hydroxyacyl-CoA dehydrogenase (EC 1.1.1.35)
Acetyl-CoA fermentation to Butyrate	Acetyl-CoA acetyltransferase (EC 2.3.1.9)
D-gluconate and ketogluconates metabolism	6-phosphogluconate dehydrogenase, decarboxylating (EC 1.1.1.44)
D-gluconate and ketogluconates metabolism	Gluconokinase (EC 2.7.1.12)

Miscellaneous

Resistance to fluoroquinolones	DNA gyrase subunit B (EC 5.99.1.3)
Resistance to fluoroquinolones	DNA gyrase subunit A (EC 5.99.1.3)
Tungstate strays	Tungsten-containing formaldehyde:ferredoxin oxidoreductase
Tungstate strays	Tungsten-containing aldehyde:ferredoxin oxidoreductase (EC 1.2.7.5)
Auxin biosynthesis	Tryptophan synthase alpha chain (EC 4.2.1.20)
Auxin biosynthesis	Anthranilate phosphoribosyltransferase (EC 2.4.2.18)
Auxin biosynthesis	Tryptophan synthase beta chain (EC 4.2.1.20)
Auxin biosynthesis	Phosphoribosylanthranilate isomerase (EC 5.3.1.24)
Nitrosative stress	Hydroxylamine reductase (EC 1.7.-.-)
SpoVS protein family	SpoVS-related protein, type 2
Quinate degradation	3-dehydroquinate dehydratase II (EC 4.2.1.10)
Thioredoxin-disulfide reductase	Thioredoxin reductase (EC 1.8.1.9)
